# The Vocal Repertoire of Gray‐Cheeked Mangabeys (*Lophocebus albigena*) in the Bugoma Forest Reserve, Uganda

**DOI:** 10.1002/ajp.70204

**Published:** 2026-09-02

**Authors:** Alexia Spelat, Arua Yolam, Thibaud Gruber, Catherine Hobaiter, Gal Badihi

**Affiliations:** ^1^ Centre for Comparative and Evolutionary Psychology, School of Psychology, Sport and Health Sciences University of Portsmouth Portsmouth UK; ^2^ Department of Animal and Human Ethology University of Rennes Rennes France; ^3^ Bugoma Primate Conservation Project Hoima Uganda; ^4^ Swiss Center for Affective Sciences and Faculty of Psychology and Educational Sciences University of Geneva Geneva Switzerland; ^5^ School of Psychology and Neuroscience University of St Andrews St Andrews UK; ^6^ Cooperative Evolution Laboratory, Deutsches Primatenzentrum GmbH Göttingen Germany; ^7^ Cooperative Cultures Research Group, Max Planck Institute for Evolutionary Anthropology Leipzig Germany; ^8^ Department of Human Origins Max Planck Institute for Evolutionary Anthropology Leipzig Germany

**Keywords:** acoustic structure, arboreal primates, call function, Cercopithecidae, communication

## Abstract

Vocal communication is central for regulating social interactions among primates, particularly in dense forest environments with limited visibility. The acoustic structure of vocalizations is often shaped by a species' socio‐ecology. However, numerous species remain poorly documented, including the gray‐cheeked mangabey (*Lophocebus albigena*), an arboreal primate endemic to the forests of Central and East Africa. We combined acoustic measurements with behavioral observations to provide a first quantitative and contextual description of the gray‐cheeked mangabey vocal repertoire in the Bugoma Central Forest Reserve (Uganda). Using a discriminant function analysis, we report four acoustically distinct call types: chuckles, grunts, screams, and whoop‐gobbles, which were also observed in different social contexts, suggesting a discrete, yet small, vocal repertoire. The two most frequently observed call types were chuckles and grunts. Chuckles were mainly produced during resting phases, and in vocal exchanges between distant individuals, suggesting they function to maintain group contact at medium or longer ranges. Grunts, in contrast, were associated with feeding and grooming, and often produced in short‐distance choruses, supporting the hypothesis that they facilitate group cohesion on a smaller spatial scale. The two other call types were more rarely observed: screams were associated with agonistic interactions, while whoop‐gobbles were produced more spontaneously, with no clear behavioral correlate that we could record, but may serve functions related to long‐distance communication. These findings provide new insights into a little‐studied species and pave the way for phylogenetic and functional comparisons with other cercopithecid primates. Nonetheless, many questions remain open and would benefit from further investigation.

## Summary

- First quantitative description of *Lophocebus albigena*'s vocal repertoire. We report four acoustically distinct call types (chuckles, grunts, screams, whoop‐gobbles) using discriminant analysis.
- Call types were associated with different behavioral contexts, suggesting distinct communicative functions linked to maintaining group cohesion at different spatial scales and showing some continuity with closely related species.
- By adding new data on a little‐studied species, this study broadens understanding of primate communicative diversity and provides a basis for comparative, evolutionary, and phylogenetic analyses.

## Graphical abstract

We describe four acoustically and contextually distinct call types in the vocal repertoire of a previously unstudied population of gray‐cheeked mangabeys. These findings provide novel data on an understudied species, expanding our understanding of primate communicative diversity and paving the way for future comparative studies.

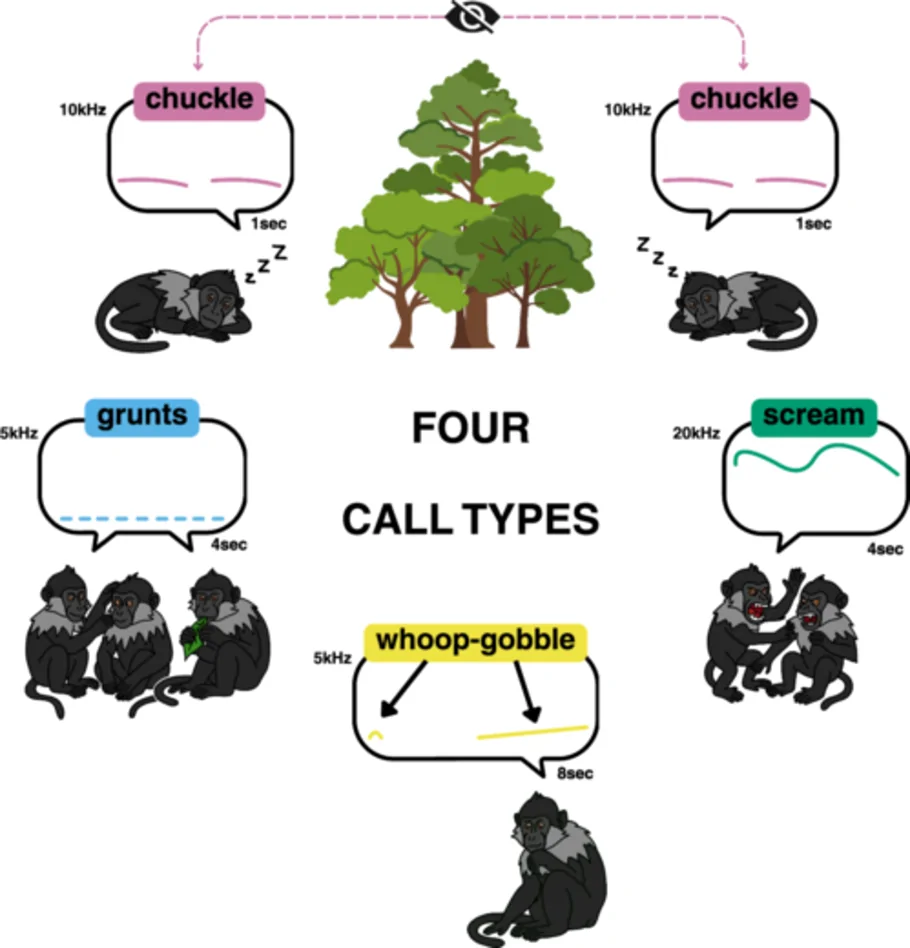

**Abbreviations**
BCFR: Bugoma Central Forest Reserve
BPCP: Bugoma Primate Conservation Project
DFA: discriminant function analysis
F0: fundamental frequency
Hz/kHz: Hertz/Kilohertz
LDA: linear discriminant analysis
n: sample size
p: probability value (*p*‐value)
SCHCC: Social Complexity Hypothesis for Communicative Complexity
s: seconds
SI: supplementary information
SD: standard deviation
VIF: Variance Inflation Factor

## 1 Introduction

Communication, in all its forms, constitutes a fundamental pillar of social interactions in animal species, allowing for the coordination of behaviors, the regulation of social relationships, and adaptation to environmental constraints (Freeberg 2022; Noë 2006; Brown et al. 1995). It is particularly crucial for group‐living species, such as primates, who face a wide variety of interactions and social partners that require similarly sophisticated capacities for the transmission of information and the expression of diverse emotional and motivational states (Freeberg et al. 2012b; Ord and Garcia‐Porta 2012). The primate order includes a large variety of species living in diverse ecosystems and different social structures. The diversity of sociality and ecology in the primate order makes this a good system in which to investigate how variation in selective pressures shapes communication. A first step in achieving this goal is to describe communicative repertoires across a phylogenetically wide range of species and populations.

Among the various channels of communication, vocal communication plays a key role in primates, including in social functions, such as maintaining social cohesion (Dunbar 1993), alerting group members to predators (e.g., Seyfarth et al. 1980; Zuberbühler 2000), reinforcing and signaling dominance relationships (e.g., Kitchen et al. 2003), and helping to maintain social bonds (e.g., Kulahci et al. 2015). The physical and ecological constraints of each environment influence the communicative modalities favored by different species. Vocal communication, which can travel across large distances and is robust to visual obstacles, is a particularly adaptive tool in environments with reduced visibility, such as dense forest canopies (Lemasson et al. 2022; Brown et al. 1995). Brown et al. (1995) demonstrated that monkeys living in tropical forests primarily use the auditory channel, which has favored the evolution of vocalizations resistant to the acoustic degradation caused by dense vegetation. The calls of these forest‐living species, such as blue monkeys (*Cercopithecus mitis*) and gray‐cheeked mangabeys (*Lophocebus albigena*), showed significantly less distortion both in time and frequency when broadcast in forested environments, as compared to when the same calls were broadcast in savannas. In contrast, savanna‐living species, such as vervet monkeys (*Cercopithecus aethiops*) and yellow baboons (*Papio cynocephalus*), did not show such habitat‐specific adaptation. Their calls showed similar, or even greater, distortion in the savanna as compared to forest habitats, suggesting that the acoustic structure of their vocalizations has not been shaped by strong selective pressures to resist habitat‐induced auditory degradation.

The evolution of vocal communication systems results from multiple interacting selection pressures, resulting in communication systems that are attuned to specific socio‐ecological contexts (Brown et al. 1995; Freeberg 2006; Lemasson et al. 2022). In this framework, comparative approaches investigating closely‐related species living in contrasting socio‐ecological environments are particularly relevant for identifying the mechanisms underlying the evolution of vocal systems (Lemasson et al. 2022).

The gray‐cheeked mangabey (*Lophocebus albigena*) represents a particularly interesting model for studying vocal adaptations. This predominantly arboreal species only rarely descends to the forest floor to forage on specific foods (A.Y. personal observations). *L. albigena* is endemic to the tropical forests of Central and East Africa. While originally placed in the genus *Cercocebus*, which includes sooty mangabeys (*C. atys)* and red‐capped mangabeys (*C. torquatus)*, the gray‐cheeked mangabey is ecologically distinct—particularly in its arboreality (red‐capped and sooty mangabeys are largely terrestrial; McGraw and Fleagle 2006) and gray‐cheeked mangabey are now formally recognized as belonging to a separate genus (*Lophocebus*) (Barnicot and Hewett‐Emmett 1972; Cronin and Sarich 1976; Disotell 1996; Fleagle and McGraw 1999; Range and Fischer 2004). In fact, *Lophocebus* is most closely related to *Papio* (baboons) and *Theropithecus* (geladas), although both *Papio* and *Theropithecus* are predominantly terrestrial (Groves 2007). As a result, the largely arboreal lifestyle of *L. albigena* is distinct from other closely related species. Unlike their terrestrial relatives, the dense forest canopies in which gray‐cheeked mangabeys live are environments in which visibility is limited and acoustic signals degrade quickly. Consequently, gray‐cheeked mangabeys may be under particularly strong selective pressure to develop call types that are acoustically distinct from one another (Marler 1965, 1967, 1973, 1976), as such discrete systems can reduce ambiguity and promote reliable communication across dense vegetation.

Despite clear potential as a model system for comparative research, the vocal repertoire of *Lophocebus albigena* remains understudied. Although some specific calls have been described in terms of their function or transmission properties, notably the whoop‐gobbles (Waser 1975, 1977; Waser and Waser 1977), the only attempt to describe a complete vocal repertoire remains that of Chalmers (1968). Based on qualitative field observations conducted in the Mabira and Lwamunda Forest Reserves in Uganda, Chalmers described five broad types of vocalizations: grunts, chuckles, screams, barks, and whoop‐gobbles. An important next step for a comprehensive understanding of the gray‐cheeked mangabey's vocal repertoire requires the addition of observations from new populations across the species' geographic range. Furthermore, building on this foundational description, quantitative approaches can be used to test whether the suggested call categories are acoustically distinct, reducing the subjectivity inherent to classification from visual spectrograms (Peckre et al. 2019).

We provide a first quantitative and contextual description of the vocal repertoire of gray‐cheeked mangabeys from a previously unstudied population in the Bugoma Central Forest Reserve, Uganda. This work extends previous studies conducted in other Ugandan forests and is guided by two main objectives: (1) to classify the types of calls produced using objective acoustic measures (duration, fundamental frequency, number of elements) and quantitative statistical methods, and (2) to document the social and behavioral contexts of vocal production to infer the potential communicative functions of call types. Because our study focuses on a single population for which detailed information on social structure and complexity is unavailable, our predictions are based primarily on ecological theories addressing signal transmission in dense forest habitats. We therefore predict that (1) the vocal repertoire of gray‐cheeked mangabeys should consist of acoustically discrete call types, and (2) call types should differ in their patterns of association with particular behavioral contexts, suggesting functional differentiation within the vocal repertoire. These analyses provide new insights into gray‐cheeked mangabey vocal repertoire and the communicative functions of specific call types, paving the way for robust interspecific comparisons and testing evolutionary hypotheses about signal structure and usage (Maynard Smith and Harper 2003; Maciej et al. 2013; McComb and Semple 2005).

## 2 Materials and Methods

### 2.1 Ethical Statement

Ethical permission to conduct the study was granted by the University of St Andrews Animal Welfare and Ethics Committee (United Kingdom) under approval code PS18274, and was approved by the Uganda Wildlife Authority (permit number: COD/96/05) and the Uganda National Council for Science and Technology (permit number: NS911ES). All data collection protocols were strictly observational and complied with the American Society of Primatologists' Principles for the Ethical Treatment of Non‐Human Primates (American Society of Primatologists 2021) and the Code of Best Practices for Field Primatology (MacKinnon et al. 2014).

To limit the risk of disease transmission between the researchers and the gray‐cheeked mangabeys, a 5‐day quarantine period was observed upon arrival in Uganda. During fieldwork, observation distances of at least seven meters were respected, face masks were worn, and hands were regularly disinfected to limit human impact and disease transmission. Environmental responsibility was also prioritized: all equipment was transported back to France or the United Kingdom for recycling, and in line with the “leave no trace” principle, all materials brought into the forest were removed after each visit. Any waste found on site was collected and properly disposed of to support the conservation of the Bugoma Forest Reserve. Finally, this study was carried out in close collaboration with local communities, including field assistants from the Bugoma Primate Conservation Project (BPCP, www.bugomaprimates.com), and we aimed to prioritize inclusivity and meaningful local engagement throughout the research process (Badihi et al. 2026).

### 2.2 Subjects and Study Site

This study was conducted in the Bugoma Central Forest Reserve (BCFR), located in northwestern Uganda. The reserve lies adjacent to Lake Albert, which forms the natural border between Uganda and the Democratic Republic of Congo. It covers an area of over 400 km^2^ of medium‐altitude semi‐deciduous tropical rainforest, ranging between 990 and 1300 m above sea level (located at 01°15' N, 30°58' E). The habitat consists mainly of dense secondary forest interlaced with seasonally flooded rivers, swamps, woodland patches, and grasslands. The reserve is situated between two other ecologically significant forest blocks: Budongo Forest Reserve (425 km^2^) and Kibale National Park (776 km^2^). Bugoma Forest hosts a particularly rich fauna, including eight species of primates, among which is the northernmost known population of gray‐cheeked mangabeys (*Lophocebus albigena*) in Uganda. These mangabeys have also been proposed to comprise several distinct species, with Groves (2007) recognizing *Lophocebus albigena*, *L. johnstoni*, *L. osmani*, and *L. ugandae*. However, this distinction remains debated, and they are currently maintained at the subspecies level (Olupot 2020). Although the exact population size within the reserve remains unknown, regular observations of wild groups have been undertaken as part of the BPCP, which has conducted long‐term monitoring of primates in the BCFR since 2016.

### 2.3 Data Collection

Before the start of formal data collection, AS, AY, and GB spent 2 weeks in the field calibrating the study protocol and aligning their observations to ensure inter‐observer consistency. Following this, we collected data on 26 days, between January 22 and March 19, 2025. Each field day AS and AY followed a group of mangabeys habituated to human presence within a maximum sampling window of 06:30–16:30, cross‐checking all observations to ensure consistency and accuracy. Once individuals from a group were located, we initiated both audio recordings and behavioral observations. In line with previous studies that identified high levels of within‐group spatial dispersion (Waser and Floody 1974), the number of individuals that we observed together varied within and across days similarly to fission‐fusion dynamics reported in other primate species (Amici et al. 2008). Therefore, we use the term “subgroup” size when referring to estimates of the number of individuals observed during a vocal event. While some individuals were recognized and observed on most observation days, as we could not identify all individuals, we cannot be certain if our observations were restricted to one or more social groups within the population, and the full size and composition of the study group(s) could not be determined.

**FIGURE 1:**
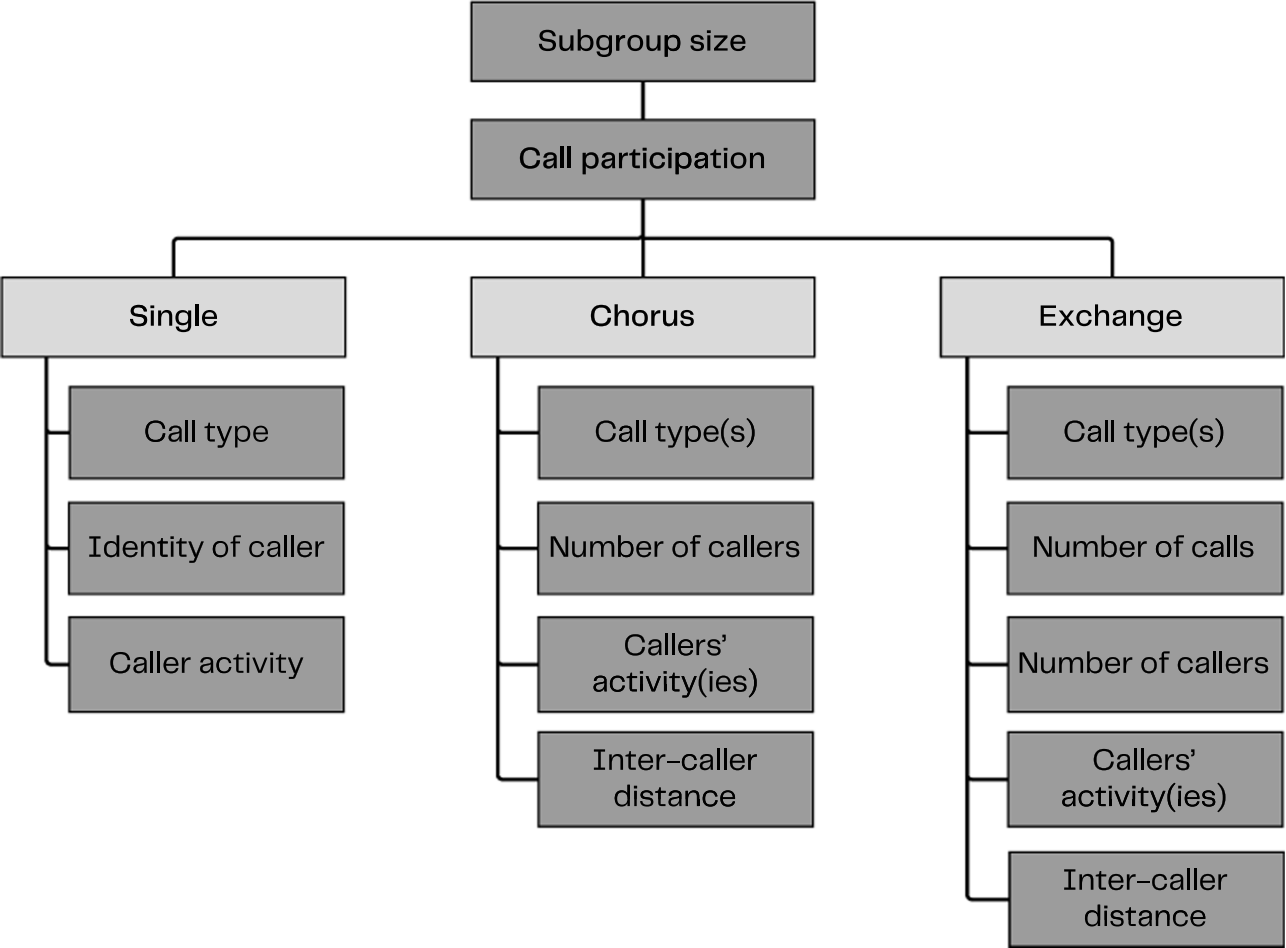
Conditional structure of vocal data collection based on call participation.

We recorded vocalizations continuously using a Marantz PMD600 recorder (sample rate 44.1 kHz, resolution 32 bits, .wav format), connected to a Sennheiser MKH416 directional microphone. Behavioral data were recorded using the software Cybertracker (https://www.cybertracker.org), installed on an iPhone 14 Pro. We used an *all‐occurrences sampling* method to record contextual information for each vocalization detected (i.e., an acoustic event). As individuals could not be reliably identified, and visibility of the callers was often limited, *focal‐animal sampling* was not feasible. When a call was heard, we collected specific variables depending on the participation of individuals in call production. We distinguished three types of participation in calling: single, chorus, or exchange. Single referred to a call produced by a single emitter, chorus involved overlapping vocalizations from two or more individuals, and exchange referred to temporally close, yet distinct (i.e., not overlapping), calls produced by two or more individuals. “Temporally close, yet distinct calls” were judged instantaneously by the observer and conformed to previously described turn‐taking patterns, with intervals of up to 5 s (Pika et al. 2018). The temporal proximity of these calls suggests that they may represent potential vocal exchanges between individuals, although this was not formally tested. The term ‘exchange’ is therefore used throughout the manuscript as a descriptive label for this pattern of call participation; however, whether these represent turn‐taking behavior remains unclear. The distinction between chorus and exchange was based on the temporal relationship between calls (overlapping vs. non‐overlapping), and not on the number of callers involved. For all types of call participation, we recorded the signalers' subgroup size, the call type(s), and the caller(s)' activity(ies). In addition, we estimated the number of callers and inter‐caller distance for both chorus and exchange calls, we also recorded the number of calls heard for exchanges, and the identity of the caller (estimated age and sex) for single calls. Inter‐caller distances were estimated in the field using the forest's trail grid system, composed of regularly spaced trails, allowing the researchers to mentally map the positions of individuals and provide reliable distance estimates, even over relatively large distances. The conditional structure followed during data collection is illustrated in Figure 1, and we provide a full description of all variables in Table 1. Call types were first defined based on the categories proposed by Chalmers (1968) and included: chuckles, grunts, screams, whoop‐gobbles, and barks. However, we did not record any instances of the “bark” call. We retained the term “chuckle,” although this vocalization has also been described as “staccato bark” in previous studies (Waser 1977; Waser and Waser 1977).

**TABLE 1:** Description of the variables collected during the all‐occurrences sampling of vocalizations.

Variable	Description	Possible values
Subgroup size	Estimated size of the subgroup present at the time of the vocalization	1, 2–5, 6–10, 11–15, > 15, unknown
Call participation	Type of vocal participation heard	Single, chorus, exchange
Call type	Type(s) of call(s) produced	Chuckle, grunt, whoop‐gobble, scream, other, unknown
Identity of caller	Age and sex of the caller (subadults were considered as mature, while juveniles and infants were classified as immature)	Mature male, mature female, mature unknown, immature, unknown
Number of callers	Number of different individuals who produced a call	Free input
Number of calls	Number of calls exchanged between callers	1, 2, 3, 4, 5, 6, 7, 8, 9, 10, > 10, unknown
Caller activity	Activity(ies) of the caller(s) at the time of the vocalization	Feeding, playing, resting, self‐grooming, allogrooming, moving, social affiliative, social agonistic, mating, other, unknown
Inter‐caller distance	Estimated average distance between callers	< 25 m, 25–50 m, 51–100 m, 101–150 m, 151–200 m, 201–250 m, > 250 m, unknown

### 2.4 Data Extraction

**FIGURE 2:**
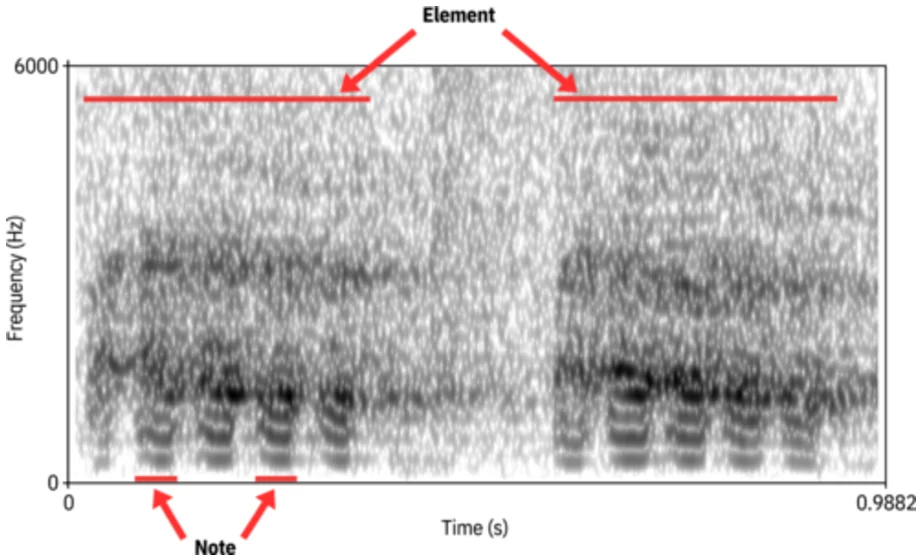
Illustration of element and note segmentation on a spectrogram of a chuckle call produced by an unidentified individual.

**FIGURE 3:**
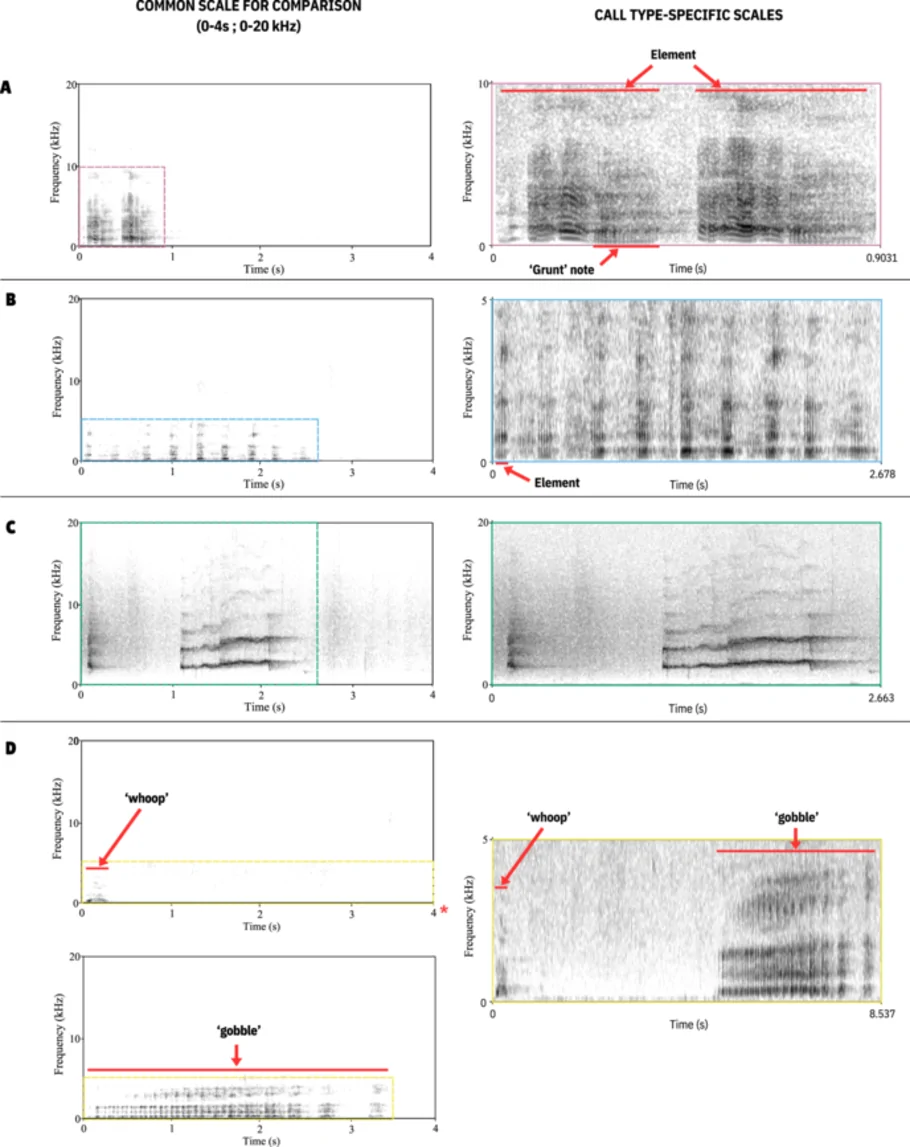
Spectrograms generated with Praat showing four types of calls: (A) Chuckle produced by a mature individual of unknown sex, (B) Grunts produced by a mature male, (C) Scream produced by a mature individual of unknown sex, (D) Whoop‐gobble produced by an unidentified individual. The left column shows all call types displayed using a common time and frequency scale (0–4 s; 0–20 kHz) to facilitate visual comparison of their overall temporal and spectral characteristics. The right column shows the same calls displayed using call type‐specific time and frequency scales to highlight their acoustic structure. *For the common‐scale spectrograms of the whoop‐gobble (D), approximately 0.9 s of pause separating the “whoop” and “gobble” elements was omitted to allow both elements to be displayed within the common 4 s time window.

To identify when vocalizations occurred on recordings, the time at which vocalizations were heard was recorded in CyberTracker (*n* = 2444) and matched with the continuous audio recordings collected in the field. We then selected vocalizations based on their acoustic quality: we only retained those that were sufficiently clear, free from overlapping noise or interference, and of high enough quality to allow reliable visualization of the fundamental frequency (F0) and other acoustic features on spectrograms. This selection process resulted in a final subset of 167 vocalizations suitable for acoustic analysis. This subset consists of an acoustically representative sample of the larger (*n* = 2444) dataset of calls recorded in this study and was used for acoustic analysis to determine whether the descriptive categories represented quantitatively and acoustically discrete calls. Acoustic analysis was conducted using Praat (Boersma and Weenink 2013). For each selected call, we extracted the following parameters: total call duration (seconds), mean fundamental frequency (F0; Hertz), F0 range (F0 max – F0 min; Hertz), number of elements within the call, and the mean duration of these elements (seconds). We defined an “element” as a continuous sound unit without any audible pause, corresponding to a breath‐like or isolated vocal emission, perceived auditorily as a distinct segment by the coder. An element may be subdivided into multiple “note”, which were defined as continuous traces on the spectrogram, separated from one another by visible gaps in time or frequency (Figure 2). However, elements and notes can sometimes be identical (e.g., grunts may also appear as individual notes at the end of a chuckle; Figure 3A).

To ensure inter‐observer reliability, GB coded a random sample of 18 (11%) of the 167 vocalizations initially coded by AS. Each call type was represented in this sample at least twice. Inter‐observer reliability was calculated with Interclass Correlation (ICC) using the *ICC* function in the R packages “psych” (Revelle 2025) and revealed good to excellent reliability across all variables (total call duration = 1; minimum F0 = 0.78; maximum F0 = 0.9, mean F0 = 0.86; number of elements = 1; mean duration of elements = 0.99).

### 2.5 Data Analysis

All analyses were conducted using RStudio software (R Core Team 2024), using the packages “dplyr” (Wickham et al. 2023), “tidyr” (Wickham et al. 2024), “purr” (Wickham and Henry 2023), “ggplot2” (Wickham 2016), “MASS” (Venables and Ripley 2002), “car” (Fox and Weisberg 2019) and “boot” (Canty and Ripley 2024).

#### 2.5.1 Discriminating Call Types Based on Acoustic Properties

To verify the species' vocal repertoire, we performed a Discriminant Function Analysis (DFA) with the *lda* function in the “MASS” package, based on the acoustic parameters extracted via Praat. This DFA assessed the extent to which call types, as categorized by us using Chalmer's (1968) repertoire, could be discriminated based on their acoustic features. We assessed multicollinearity between variables using Variance Inflation Factors (VIF) with the *vif* function in the “car” package. This check revealed no violation of the assumptions regarding collinearity, as VIF values ranged from 1.09 to 3.84 (Table S1), which is below the typically applied threshold of 10, used to indicate problematic multicollinearity (Quinn and Keough 2002; Field 2005). However, some authors recommend more conservative thresholds of 3 or even 2 (Zuur et al. 2010). In our case, the highest VIFs suggested potential collinearity between the mean duration of elements, the total call duration, and the number of elements per call. Nevertheless, we chose to include all three variables, as we considered that while the total duration of calls is closely linked to element length in calls with few elements, a longer call could also result from a greater number of short elements rather than from longer elements, thereby making these measures theoretically distinct and informative. Similarly, potential collinearity was observed between the mean F0 and the F0 range, but both variables were also retained, as they provide complementary, yet distinct, information: the overall pitch level of the call and its pitch variability, respectively.

We built the DFA model using prior probabilities based on the actual proportions of call types recorded in the field (47.83% for chuckles, 48.60% for grunts, 1.74% for scream, 1.82% for whoop‐gobble). We chose this approach in order to avoid biases introduced by the uneven sample sizes obtained after acoustic extraction (72 chuckles, 39 grunts, 38 screams, and 18 whoop‐gobbles). Such disparities were considered to be unlikely to reflect biological differences in call usage, as compared to technical limitations during extraction (e.g., grunts, although produced very frequently, were often produced in choruses and thus more difficult to isolate for acoustic measurements). Using field‐based priors allowed the model to better approximate the natural distribution of calls. The model was then validated through a leave‐one‐out cross‐validation procedure. We tried to follow the rule of thumb of including at least 20 samples per group (call‐type) in a DFA; however, we only manage to extract 18 high‐quality whoop‐gobble recordings. Therefore, to ensure our results were robust, we calculated the 95% confidence intervals for the DFA accuracy by bootstrapping the data 1000 times using the *boot* function in the “boot” package. We also report the bias, which represents the average difference between the observed accuracy measure (the proportion of correct classifications) and the accuracy measure from the bootstrapped samples, and the standard error, which estimates the variability of the accuracy measure. For comparison, we also ran the DFA using equal prior probabilities (this analysis and results are reported in Supplementary Analysis S1: Figure S1; Tables S2–S3).

#### 2.5.2 Functional Analysis of Call Types

Once the discriminant function analysis confirmed whether our classification of call types represented acoustic discrete categories, we used the larger dataset (*n* = 2444) to investigate the impact of specific social and behavioral factors on call production using three Fisher's exact tests. We chose Fisher's tests over Chi‐squared tests because the data did not meet the assumption that the expected cell frequency should be greater than five, as required for Chi‐squared analysis. We tested for significant associations between: (i) the call type and the caller's activity, (ii) the call type and the form of vocal participation (single, chorus, or exchange), and (iii) the inter‐caller distance and the form of vocal participation.

We excluded observations containing missing values or "unknown" labels in the variables of interest. In addition, we removed observations involving calls labeled as "other" due to their low frequency (*n* = 5). For the first test (i), which involved the relationship between call type and caller activity, two ambiguous observations involving multiple call types and multiple caller activities within a single observation were further excluded, as no clear match could be established between calls and activities. After filtering, the number of acoustic events recorded on CyberTracker was reduced to *n* = 617 for test (i), *n* = 2437 for test (ii), and *n* = 1051 for test (iii). For tests (i) and (ii), where the analyses involved the variable “call type”, multiple call occurrences could be extracted from a single observation event when several call types were identified within the same observation; for example, when both chuckles and grunts were produced in a chorus or exchange. As a result, the final sample size used in the analysis (i.e., the number of call‐type‐specific entries) was larger than the number of acoustic events recorded on CyberTracker: *n* = 684 for test (i), and *n* = 2580 for test (ii). In contrast, test (iii) did not use the “call type” variable and was therefore performed on a one‐row‐per‐event basis (*n* = 1051). We computed expected frequencies under the assumption of independence and examined deviations using standardized residuals. The significance threshold for all statistical tests was set at *α* = 0.05.

## 3 Results

### 3.1 Dataset Description

The final dataset included a total of 2444 acoustic events recorded on CyberTracker. Among these, single calls were the most frequent (*n* = 1374), followed by call exchanges between individuals (*n* = 607), and call choruses (*n* = 463). Most single calls were attributed to unidentified individuals (*n* = 1159), followed by mature individuals of undetermined sex (*n* = 106). Due to the dense canopy, callers could only be reliably identified in 109 acoustic events: 44 mature females, 38 immature individuals, and 27 mature males. Across all call types, the most commonly observed were grunts (*n* = 1254) and chuckles (*n* = 1234). The other call types were substantially less frequently observed: whoop‐gobble (*n* = 47), screams (*n* = 45), and atypical or uncategorized calls (*n* = 5).

### 3.2 Description and Discrimination of Call Types

Chuckles were recorded 621 times as single calls, 519 times during exchanges, and 94 times during choruses. These calls were audible over long distances, with maximum distances estimated at greater than 500 m, and were emitted by individuals from all age and sex categories, including 28 observations from adult females, 10 from adult males and 24 from immature individuals (unknown age/sex: *n* = 495; mature unknown: *n* = 64). Acoustically, chuckles were relatively high‐pitched and showed a wide frequency range. These calls were composed of one or more elements emitted in quick succession. Most commonly, these calls were made up of two (Figure 3A) or a single element(s). On average, these vocalizations were short, lasting less than 1 s, and were composed of short elements (Table 2). However, we also observed longer variants comprising more than 10 consecutive elements. This variability in sequence length was also found within individual elements, which included between 3 and 10 successive notes (Figure 3A).

**TABLE 2:** Mean ± standard deviation of acoustic parameters extracted with Praat for each type of call (72 chuckles, 39 grunts, 38 screams, and 18 whoop‐gobbles).

Acoustic parameters	Chuckle	Grunts	Scream	Whoop‐gobble
F0 mean	407.7 ± 195	81.5 ± 29	3278.2 ± 828.2	286.9 ± 33.1
F0 range	202 ± 144	34.6 ± 57.6	1832 ± 1072.2	226.7 ± 50.5
Total duration	0.8 ± 0.6	3.7 ± 2.1	4.1 ± 3.6	8 ± 0.8
Number elements	1.8 ± 0.9	11.8 ± 7.8	5 ± 4.7	2 ± 0
Mean duration elements	0.4 ± 0.2	0.1 ± 0.03	0.6 ± 0.3	2 ± 0.6

We also observed grunts in all three vocal participation modes: 667 times as a single call, 457 times during choruses, and 130 during exchanges. Adult males (*n* = 14) and females (*n *= 10) were observed producing these calls, but no occurrences were recorded from immature individuals (unknown age/sex: *n* = 604; mature unknown: *n* = 39). Grunts had a more fragmented structure, composed of many very short elements, forming sequences with apparently evenly‐spaced intervals (Figure 3B). Grunts lasted 3.7 s on average and were lower‐pitched, quieter, and generally only audible at short range (estimated at around 50 m) as compared to chuckles (Table 2). Our qualitative observations suggest that these vocalizations are frequent within the group, particularly during periods of resting or feeding. Grunts also accompanied social approaches or followed brief episodes of agonism in the group.

Screams were less frequent (*n* = 45) than grunts and chuckles and were mostly recorded as single calls (*n* = 43), with fewer instances in choruses (*n* = 1) or during exchanges (*n* = 1). They were primarily produced by immature individuals (*n* = 14), but also occasionally by adult females (*n* = 5) and males (*n* = 2), during aggressive interactions (unknown age/sex: *n* = 21; mature unknown: *n* = 1). Screams were high‐pitched and had a wide frequency range (Figure 3C). On average, they were 4.1 s in duration, and contained five elements, each lasting 0.6 s (Table 2).

Like screams, whoop‐gobbles were also relatively rare (*n* = 47) as compared to grunts and chuckles. These were loud calls that could be heard over long distances, estimated to exceed 600 m. They were recorded as single calls in 38 cases, and rarely in choruses (*n* = 1) or during exchanges (*n* = 8). Although most of these calls were produced by individuals who could not be identified (*n* = 36) because we were too far away from the caller, we directly observed one adult male and one adult female producing them. These calls consist of an initial brief whoop element (*n* = 18; mean duration = 0.2 s ± 0.04), followed after a short pause averaging 4 s ± 1.5, by a longer phase referred to as the gobble (mean duration = 3.7 s ± 1.2; Figure 3D). They stand out due to their longer overall duration, as compared to other call types and a discrete structure comprising two different element types. They had a moderate pitch (whoop: mean F0 = 289.9 Hz ± 48.8; gobble: mean F0 = 283.9 Hz ± 29.7) and exhibited a substantial frequency range (Table 2).

Audio examples of the four call types (chuckles, grunts, screams, and whoop‐gobbles), corresponding to the spectrograms shown in Figure 3, are provided in the Supplementary Information (Audio S1–S4).

**FIGURE 4:**
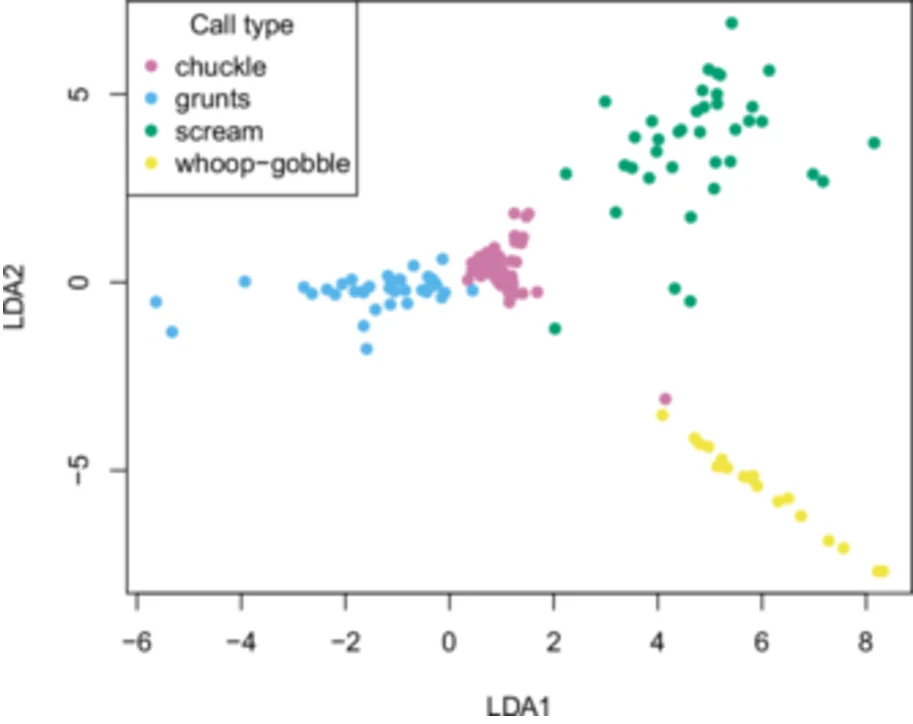
Projection of vocalizations onto the first two canonical variates (LDA1 and LDA2); each point represents a call.

The four call types were acoustically distinct, allowing for their successful classification using discriminant function analysis (DFA) of 5 acoustic features (92.8% correct classification rate). The two main discriminant canonical variates extracted by the DFA accounted for 62.7% (LDA1) and 21.6% (LDA2) of the discriminatory power, respectively (hence, reaching 84.3% of the between‐group variability explained in total). The projection of vocalizations onto these two axes showed clear separation between all four call types (Figure 4).

The first canonical variate (LDA1), which accounted for most of the discriminatory power, was primarily driven by the mean duration of elements and the total duration of the calls, both of which loaded positively (1.9211 and 0.2972, respectively). This axis thus appears to reflect a temporal dimension, distinguishing longer, less segmented vocalizations with shorter, more fragmented ones (such as grunts). The second canonical variate (LDA2) also represented the mean duration of elements and the total duration of the calls, but here both variables loaded negatively (−1.8596 and −0.3733, respectively). Although driven by the same temporal parameters, LDA2 captured a different aspect of variation, contributing in particular to the separation of whoop‐gobbles, which tended to have longer overall durations.

The coefficients of the linear discriminants for each acoustic variable in the first two canonical variates (LDA1 and LDA2) are presented in Table 3.

**TABLE 3:** Coefficients of the linear discriminants for each acoustic variable in LDA1 and LDA2.

Variable	LDA1	LDA2
F0 mean	0.0010	0.0015
F0 range	0.0001	0.0003
Duration	0.2972	−0.3733
Number elements	−0.2394	0.0596
Mean duration elements	1.9211	−1.8596

The performance of the model was evaluated using leave‐one‐out cross‐validation. The overall correct classification rate was 92.8% (95% confidence intervals = 90.4–97.6%, bias = 1.14%, standard error = 2.1%), indicating high precision even within the limited sample of whoop‐gobbles. The confusion matrix resulting from this cross‐validation is presented in Table 4.

**TABLE 4:** Cross‐validated confusion matrix obtained through leave‐one‐out cross‐validation.

	Chuckle	Grunts	Scream	Whoop‐gobble	Sum
Chuckle	71	0	0	1	**72**
Grunts	9	30	0	0	**39**
Scream	2	0	36	0	**38**
Whoop‐gobble	0	0	0	18	**18**
Sum	**82**	**30**	**36**	**19**	**167**

Most chuckles and screams were correctly classified, and all whoop‐gobbles achieved a 100% successful classification rate. However, some grunts were misclassified as chuckles, which represents the main source of classification error (*n* = 9).

### 3.3 Behavioral and Social Functions of Call Types

#### 3.3.1 Caller Activity During Vocal Production

We recorded the caller's behavior for a total of 617 acoustic events, resulting in 684 call‐type‐specific entries. These were mainly composed of chuckles (*n* = 341) and grunts (*n *= 319), as well as a smaller number of screams (*n* = 17) and whoop‐gobbles (*n *= 7). We found a strong association between call type and caller activity (Fisher's exact test; *p* = 0.0005).

**FIGURE 5:**
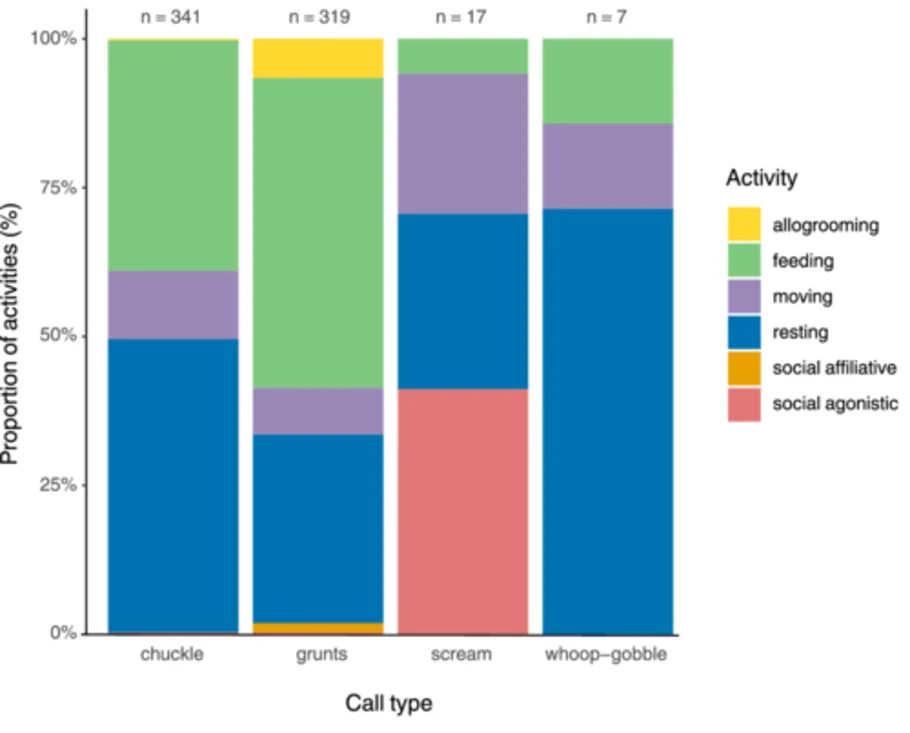
Stacked bar charts illustrating the proportional distribution of caller activities across call types (341 chuckles, 319 grunts, 17 screams, and 7 whoop‐gobbles).

Overall, calls were most frequently produced during resting and feeding (Figure 5). Chuckles were most likely to be associated with resting (standardized residual = +2.5), and were less likely to be produced during allogrooming (standardized residual = −3.0). Conversely, grunts were predominantly recorded during allogrooming (standardized residual = +3.4) and feeding (standardized residual = +2.2), and were less likely to be observed during resting as compared to other calls (standardized residual = −2.6). Screams were most likely to be associated with agonistic interactions (standardized residual = +14.3), whereas whoop‐gobbles showed no strong association with any particular activity, with residuals close to zero. The table of standardized residuals can be found in the Supplementary Information (Table S4).

#### 3.3.2 Call Participation Across Call Types

**FIGURE 6:**
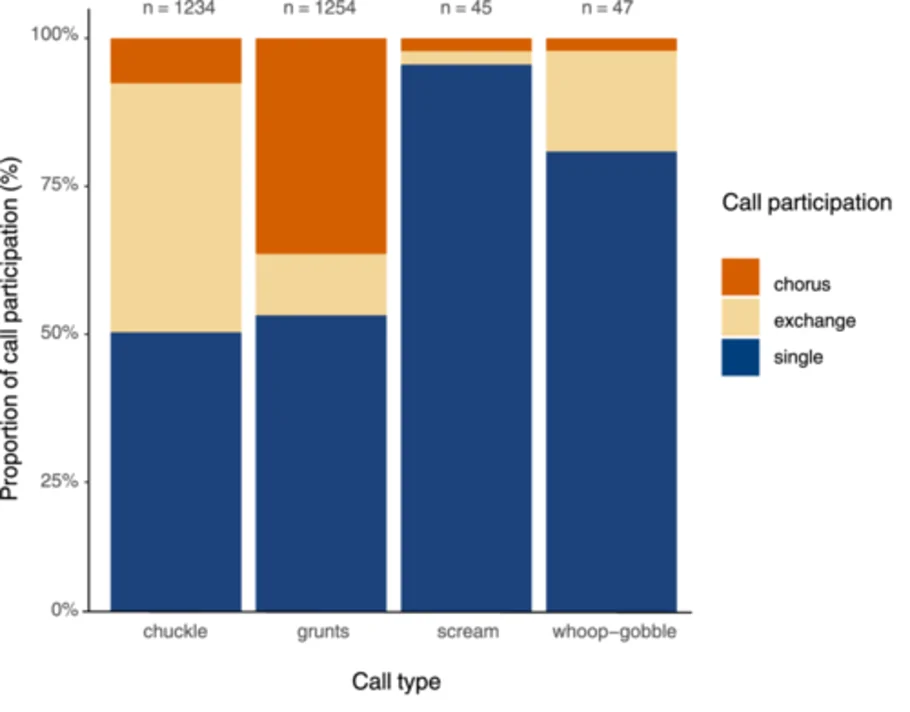
Stacked bar charts illustrating the proportional distribution of call participation across call types (1234 chuckles, 1254 grunts, 45 screams, and 47 whoop‐gobbles).

Among the calls that could be extracted for further analysis (*n* = 2580), 1369 were produced as single calls, 658 as part of vocal exchanges between individuals, and 553 in a chorus. Different call types were more likely to be associated with specific types of participation (Fisher's exact test; *p* = 0.0005; Figure 6).

Across call types, the majority were produced as single calls. However, chuckles were less likely to be produced in choruses (standardized residual = −10.5) and more likely to be produced in exchanges (standardized residual = +11.5), with approximately 42% of chuckles occurring in exchanges. In contrast, grunts were most likely to be produced in choruses (standardized residual = +11.5), which was the case for about 36% of the grunts recorded. Screams and whoop‐gobbles were more frequently observed to be produced as single calls (standardized residuals of +3.9 and +2.6, respectively). Standardized residuals for all call‐participation associations are reported in the Supplementary Information (Table S5).

#### 3.3.3 Inter‐Caller Distances in Multi‐Caller Call Participation

The analysis of inter‐caller distances was restricted to vocalizations produced in chorus or exchange contexts, for which this information was available. This included a total of 1051 acoustic events, composed of 599 exchanges and 452 choruses. Inter‐individual distance varied with the type of call participation (Fisher's exact test; *p* = 0.0005).

**FIGURE 7:**
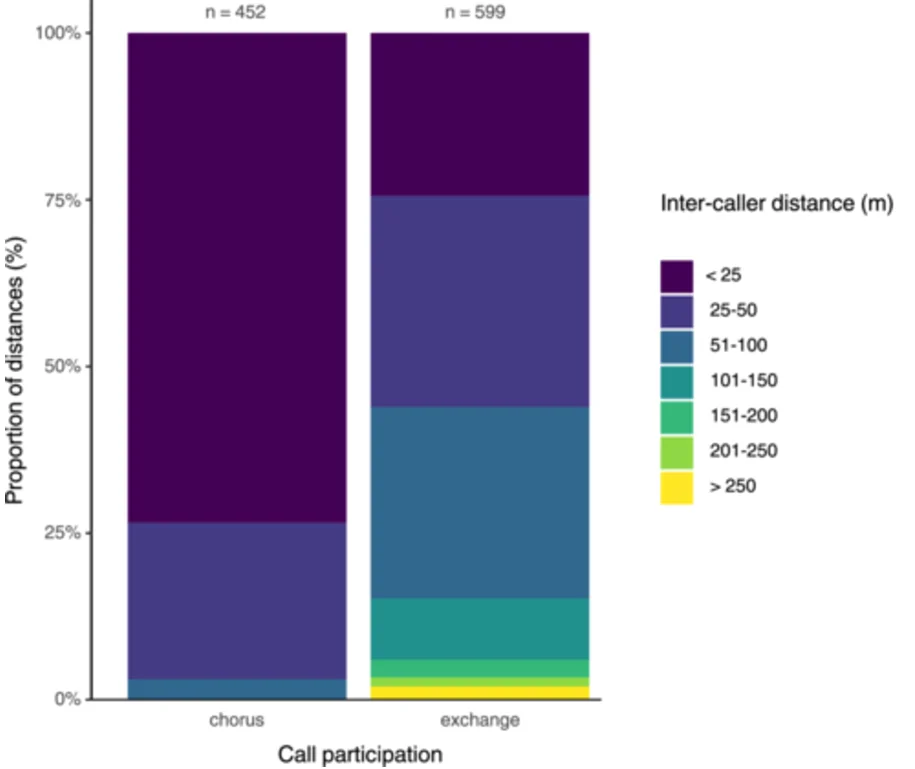
Stacked bar charts illustrating the proportional distribution of inter‐caller distances by call participation mode (452 chorus and 599 exchanges).

In general, chorus vocalizations were more likely to be observed when individuals were in close proximity, especially at distances below 25 meters (standardized residual = +8.8). Conversely, exchanges were more likely to be observed at greater distances, particularly in the 51–100 m (standardized residual = +6.4) and 101–150 m (standardized residual = +4.2) ranges. A marked under‐representation of chorus vocalizations was observed in the 51–150 m range (standardized residual = −7.4 for 51–100 m; standardized residual = −4.9 for 101–150 m), while exchanges were rarely produced at distances of under 25 m (standardized residual = −7.7) (Figure 7). Standardized residuals for all call participation‐distance associations are reported in the Supplementary Information (Table S6).

## 4 Discussion

We provide the first quantitative description of the vocal repertoire of gray‐cheeked mangabeys (*Lophocebus albigena*) in the Bugoma Central Forest Reserve in Uganda, based on both acoustic parameters and the social contexts of call production. Following descriptions of call categories from other gray‐cheeked mangabey populations (Chalmers 1968; Waser 1977), we identified four major call categories in our study group(s), which were also reported in other populations: chuckles (also referred to as “staccato barks”; Waser 1977), grunts, screams, and whoop‐gobbles. These call types were acoustically distinct and observed in different behavioral contexts, suggesting functional differentiation within the vocal repertoire.

### 4.1 Repertoire

The clear acoustic separation of the four call categories identified in the Bugoma population suggests that these vocal signals are structurally discrete, facilitating reliable discrimination between call types. This pattern is consistent with theoretical predictions regarding acoustic communication in dense forest habitats, where signal degradation and limited visibility may favor the evolution of acoustically distinct vocalizations (Marler 1965, 1967, 1973, 1976). Nevertheless, the repertoire documented in this study remains smaller than those reported for other primate species, many of which are also arboreal (e.g., 19 in *Cercocebus atys*; Range and Fischer 2004; 10 in female *Cercopithecus campbelli campbelli*; Lemasson and Hausberger 2011; 6 in male *Cercopithecus mitis stuhlmanni*; Fuller 2014; 36 in *Cercopithecus aethiops*; Struhsaker 1967). Differences in vocal repertoire size between species have been linked to differences in social complexity. The social complexity hypothesis for communicative complexity (“SCHCC”) argues that animals living in socially complex groups develop more elaborate communication systems to meet the demands of their social environment (Freeberg 2006; Freeberg et al. 2012a, 2012b). While differences in social structure may explain some cross‐species differences, further descriptions of the gray‐cheeked mangabey social structure and organization across populations are required to address the social complexity hypothesis for communicative complexity in this species.

Given the relatively short study period, our observations may not have captured the full vocal repertoire of gray‐cheeked mangabeys, and previous studies reported additional vocalizations that were not documented in our dataset. For example, Chalmers (1968) described a “bark” call, typically produced by the aggressor during chasing interactions, most often by adult males. Waser (1977) noted that this call, along with several other infrequently produced vocalizations, occurred only rarely and in highly specific contexts. Importantly, these calls were not included in previous acoustic analyses, and we could not obtain detailed spectrographic descriptions. Consequently, it remains unclear whether such vocalizations were absent from the Bugoma population, occurred too infrequently to be detected during our study period, or were present and recorded but not identified as distinct call categories in our classification process. Indeed, while we could discriminate between four major call categories, there was substantial variation within call types in their acoustic features both in our dataset and in previous descriptions, suggesting the potential for call subtypes within these broad categories or a larger repertoire with gradation in acoustic features.

As in other species, screams were high‐intensity calls, audible over long distances, and characterized by acoustic heterogeneity (Owings and Morton 1998). Whoop‐gobbles were the most distinctive, and matched descriptions of previous studies as being a high‐amplitude, stereotyped call that requires the inflation of the extra‐laryngeal vocal sac to be produced (Chalmers 1968; Gautier 1971; Waser 1977). Homologous high‐amplitude calls have also been described in other mangabey species as whoop‐gobbles (*Cercocebus*: Range and Fischer 2004) or as “grating roar” and “roar grunt” in baboons (Waser 1982). The acoustic differences between these calls may reflect specific adaptations to the local environment (Range and Fischer 2004).

While screams and whoop‐gobbles appear to fit into discrete categories, we could qualitatively distinguish several acoustic subtypes among the chuckles and grunts when hearing them in the field (Table S7). In line with these observations, earlier descriptions of chuckles also reported some variability in these call categories (e.g., Chalmers 1968; Waser 1977). However, these studies linked variation in pitch to sex and age differences (e.g., Chalmers 1968) and/or suggested variation linked to the context of production (Waser 1977). In our dataset, chuckles exhibited the most structural variability of all four call types, at multiple levels (number of elements, number of notes per element, fundamental frequency F0). In some cases, we heard notes resembling a gradation towards grunts at the end of a chuckle (Audio S1) or an initial chuckle note with a higher pitch (Audio S5, Figure S2). However, because it was unclear whether this first note reflected an actual higher‐frequency or simply an inhalation preceding the vocalization, it was not included in the frequency measurements.

Grunts showed similar variation in our dataset, echoing previous studies. Chalmers (1968) described grunts as highly flexible vocalizations varying in amplitude, rate, and bout structure, and Waser (1977) subsequently distinguished between “soft” and “loud” grunts, also noting contextual differences in their production. Building on previous studies by Chalmers and Waser, Brown (1989) summarized a classification recognizing several additional variants, including “rattling grunts”, “normal chorused grunts”, “loud chorused grunts” and “postcopulatory grunts”. Consistent with these descriptions, we qualitatively distinguished several apparent grunt variants during field observations (Table S7). However, many of these calls, particularly the “soft grunts”, were produced at low amplitudes and were often difficult to record clearly under field conditions. As a result, we did not obtain enough high‐quality recordings for each variant to permit robust acoustic analyses.

Such variability within call categories suggests the existence of subtypes, similarly to those described in *Cercopithecus campbelli* (Lemasson and Hausberger 2011), which can be distinguished using several acoustic criteria (e.g., number of units, repetition rhythm, and harmonic richness). Alternatively, the fact that some chuckles ended with grunt‐like notes or were produced in combination with grunts could suggest a blurring of the boundary between the two call types, indicating a gradual continuum between chuckles and grunts. This continuum could also account for the adjacent position and slight overlap observed for these two call types in the acoustic analysis, suggesting that some of the calls produced fell into intermediate acoustic zones. As previously suggested, acoustic variability may be due to age or sex differences (Chalmers 1968) or context (Waser 1977). While our sample did not allow for a quantitative test of how acoustic properties of calls varies with age, sex, or context, this would be a fruitful focus for future studies.

### 4.2 Function

While we could not test how the acoustic properties of each call type varied with context, different call types were produced in different contexts, with different types of participation, and at different inter‐caller distances (for calling events with multiple callers). Chuckles were most often produced in vocal exchanges at medium‐range distances (50–150 m), in which callers were not in visible contact. Combined with the finding that they were most often produced in resting contexts, our observations suggest that chuckles meet the criteria for “contact call” and function to promote group cohesion by maintaining connection between conspecifics even when out of sight (Kondo and Watanabe 2009). This type of vocal interaction, generally involving only one call type from the vocal repertoire exchanged between individuals (Meunier et al. 2024), has been documented across primate species (e.g., Chow et al. 2015; Takahashi et al. 2013; Lemasson et al. 2011; Lemasson et al. 2005). Beyond merely quantifying exchanges, it is important to note that incorporating temporal information would be valuable. In fact, assessing whether these interactions follow turn‐taking rules requires examining the precise timing between callers (e.g., Demartsev et al. 2018). In our study, while exchanges between mangabeys were identified, further analysis would be needed to determine whether these represent true turn‐taking, for example by identifying overlap avoidance (Demartsev et al. 2018).

That being said, it is important to note that chuckles were also observed (more rarely) in a wide variety of contexts, including following sudden noises or vocalizations from other species (e.g., baboons), which may suggest that these calls serve additional functions or a function that is relevant across contexts. In such situations, several individuals began to vocalize simultaneously, unlike the apparent “exchanges”, suggesting a collective response to a shared stimulus. These observations also align with previous descriptions by Waser (1977), who suggested that chuckles (staccato barks) were a form of intra‐group vocal contagion, during which calls are temporally clumped, and Chalmers (1968) original interpretation of chuckles as signals for alerting group members to moderate threats. In light of this, the diversity of contexts in which chuckles are produced suggests either a multifunctional role or a role that is flexibly expressed across behavioral contexts, combining social and/or informational functions depending on the situation.

This hypothesis is supported by remarks from Brown (1989), who identified this call type as one of the most adaptable in the vocal repertoire, produced both in alarm contexts and in situations involving positional marking. Such functional versatility highlights the value of future research targeting chuckles, to refine our understanding of their structural and contextual variability.

Grunts were the most commonly recorded call type and were primarily associated with feeding or allogrooming. We also observed grunts being produced when individuals approached one another, and following aggressive interactions, as previously described by Chalmers (1968) and in other primate species (e.g., *Cercocebus torquatus atys*; Range and Fischer 2004; *Papio cynocephalus ursinus*; Rendall et al. 1999; *Cercopithecus aethiops*; Cheney and Seyfarth 1982; *Cercopithecus mitis*; Brown 1989; *Cercopithecus cephus* & *Cercopithecus nictitans*; Gautier and Gautier‐Hion 1988); however, we did not specifically record approach as a behavioral context. These interspecific convergences suggest that grunts may serve a pacifying function in mangabeys, similarly to their role in other primates (Cheney et al. 1995; Cheney and Seyfarth 1997). Chalmers (1968) also noted a correlation between the emission of these vocalizations and a reduction in agonistic behaviors, supporting the hypothesis of a calming or reassuring function. Our observations of grunts align with previous reports of these being the most common calls in other species and similar to other low‐intensity calls such as “grunts”, “coos”, or “trills” often referred to as within‐group “contact calls” (e.g., Cheney et al. 1995; Sugiura 2007; Rendall et al. 1999).

Grunts were also more likely to be produced in chorus when individuals were close together (< 25 m). Chorus calls are common in animal behavior and have been reported in other primate species (e.g., baboons; DeVore and Hall 1965; Rowell 1966; macaques; Rowell and Hinde 1962; Itani 1963). The simultaneous emission of grunts by several individuals likely increases the amplitude and reach of the signal as a whole, which is relatively low in intensity when produced in isolation. Given that gray‐cheeked mangabeys live in socially cohesive but spatially dispersed groups occupying home ranges that span several square kilometers (Waser 1975), adjusting the acoustic properties of grunts by chorusing (i.e., making them louder) may play an important role in maintaining cohesion between dispersed subgroups. Nevertheless, they may have multiple potential functions, potentially linked to the acoustic variability within this call type. In many animal species, coordinated vocalizations fulfill diverse roles, including reinforcing social bonds, defending territory, monitoring mating partners, and coordinating group activities (Geissmann 2002; Hall 2004; Méndez‐Cárdenas and Zimmermann 2009).

The screams recorded in this study match observations of the acoustic structure and function of screams in other species. Across species, including the gray‐cheeked mangabeys in this study, they are produced by individuals of all ages, and likely serve convergent functions such as recruiting support during agonistic interactions (e.g., in cercopithecid species: Gouzoules et al. 1984, 1986; Gouzoules and Gouzoules 1989; Lindburg 1971; Cheney 1977; de Waal 1977; Bernstein and Ehardt 1985), deterring a potential attacker (Owren and Rendall 1997, 2001; Rendall and Owren 2002), or to drawing attention to the caller, typically from the mother or other group members (Rendall et al. 2009).

Finally, the whoop‐gobbles were the most distinctive vocalization, both structurally and functionally. These calls were usually produced by distant, and thus unidentifiable, individuals, which limited our ability to infer their function based on direct observation. Nevertheless, our observations match reports from several other studies that have focused on this call type. In those studies, whoop‐gobbles were described as long‐distance calls produced “spontaneously” by adult males (Chalmers 1968; Waser 1977). However, in this study, we observed at least one instance of a whoop‐gobble produced by a female (accompanied by her infant) following an intra‐group aggression. To our knowledge, no other descriptions of wild female whoop‐gobbles have been published. Although Chalmers (1968) reported a single occurrence of this call by an adult female in captivity, all wild observations in that study involved adult males. We posit the tentative hypothesis that specific females may produce vocalizations more typically used by males under relevant affective or social contexts. These cases of “atypical” use may be produced as a result of stress or threat, especially when immediate adaptive benefits are at stake (e.g., protecting offspring). Alternatively, female‐use may be a typical, if relatively rare, part of their vocal behavior. In the absence of repeated observations, it remains difficult to draw firm conclusions. Our observations align with results from play‐back experiments in gray‐cheeked mangabeys (Waser 1977) and descriptions of similar calls in *Cercopithecus* species (Struhsaker 1970; Gautier 1969), which have been shown to play a role in maintaining group cohesion when individuals are dispersed. Furthermore, Waser's play‐backs (1977) suggests that whoop‐gobbles may also serve roles in individual recognition, guiding collective movement, and maintaining inter‐group spacing. More generally, powerful vocalizations in forest‐dwelling primates, such as chimpanzee pant hoots (Wilson et al. 2007), *Cercopithecus* long calls (Struhsaker 1970; Gautier 1969), and howler monkey roars (Carpenter 1934) have often been associated with intergroup or intertroop spacing functions (Carpenter 1934; Carpenter 1965).

### 4.3 Limitations

While our 3‐month study period allowed for a relatively large sample (*n* = 2444), it did not allow us to capture seasonal variation in the acoustic communication of this population of gray‐cheeked mangabeys. Furthermore, our study was conducted on a single population in the Bugoma Central Forest Reserve. Although several of the call types identified here correspond closely, in both acoustic and contextual features, to vocalizations described in other Ugandan populations, broader sampling across multiple populations will be required to assess the extent to which call structure, usage, and contextual associations are conserved throughout the species' range. Long‐term studies spanning different seasons and years, and incorporating additional populations, will therefore be necessary before these patterns can be considered representative of gray‐cheeked mangabeys more generally. Moreover, the dense secondary canopy of the study site and the species' arboreal mode of life resulted in generally low visibility for much of the time, so many contextual details were rarely available, particularly for the group as a whole. Consequently, we relied primarily on caller activity to infer the general context of calls. This approach necessarily leaves contexts broad and imprecise and omits important information, such as recipient responses. Future research should focus on more specific types of contexts, including for the group as a whole, and record recipient reactions to better clarify call function.

Some calls were heard more frequently than others, meaning that our ability to draw conclusions from our sample varies accross call types. For example, the sample size for whoop‐gobbles and screams was much smaller than that for chuckles and grunts. In terms of the acoustic analysis, the higher proportion of screams produced by immature individuals (as compared to other call types) may have introduced a potential bias in the acoustic analyses. Calls produced by younger individuals typically featured higher fundamental frequencies, which could have influenced their overall spectral profile and introduced greater variability in our data. However, this type of variability is common in screams across species and age classes (Owings and Morton 1998), suggesting it is unlikely that our findings are driven solely by the signaler's age.

Despite the behavioral and acoustic interest that grunts offer, their acoustic analysis was technically limited in this study. Their low fundamental frequency, combined with their low amplitude, made them particularly difficult to record in the noisy and dense environment of the Bugoma forest. Background noise, and the tendency for grunts to be produced in chorus made it very challenging to isolate individual calls on spectrograms (due to signal overlap), leading to an underrepresentation of grunts in the acoustic (as compared to the behavioral) dataset. This bias likely restricted the measurable variability in our acoustic analysis of grunts, which may partly explain the lack of clear differentiation in the acoustic analysis. To overcome these limitations, future studies could use recordings made in semi‐controlled conditions, such as in captivity, using individual telemetry‐based recording devices.

When considering the functions of calls, we only considered the behavior of the mangabeys that we could see, both the signalers and audience. However, we noticed that gray‐cheeked mangabeys also called in the presence of, and as a potential reaction to other sympatric primates. Most notably, chuckles were observed in response to vocalizations from, and in the presence of, red‐tailed monkeys (*Cercopithecus ascnius*), who were often observed in the same feeding tree as the gray‐cheeked mangabeys. To gain a full understanding of call functions in gray‐cheeked mangabeys, it may be productive to consider their interactions with other sympatric species.

## 5 Conclusion

This work represents an initial starting point in the quantitative study of the vocal repertoire of *Lophocebus albigena*. We describe four acoustically distinct call types that were associated with different behavioral contexts, suggesting potential functional differentiation. While grunts, chuckles, and whoop‐gobbles all appear to be broadly linked to the maintenance of group cohesion and coordination, they may do so at different spatial scales. Grunts were used in short‐distance interactions, chuckles at medium distances, and whoop‐gobbles at longer distances. As in other social species living in visibly dense habitats, maintaining auditory contact is crucial for group cohesion and coordination across spatial scales. Screams may serve a distinct function: to mediate agonistic interactions, aligning with the function of screams in other primate species. However, the functional interpretations proposed here remain tentative and require further testing. Moreover, our findings reflect only a first glimpse of the species' potential vocal diversity. Several signals, such as chuckles and grunts, exhibited substantial variability, both in form and in usage, suggesting the presence of yet under‐characterized subtypes or gradation. Many questions remain open concerning these vocalizations, and such elements deserve to be explored in greater depth through complementary approaches, and the addition of more populations and more refined methodological tools. This study opens the door to systematic comparative analyses with other members of the *Lophocebus* genus, as well as with closely related species such as baboons (*Papio*) and other mangabeys (*Cercocebus*), which appear to share many vocal similarities. By providing novel insights into a species that remains under‐studied, our findings contribute to advancing our understanding of the evolution of vocal communication and its relationship with primate social structure, ecology, and phylogeny.

## Author Contributions

**Alexia Spelat:** conceptualization, methodology, data curation, formal analysis, visualization, funding acquisition, writing – original draft, writing – review and editing, investigation, validation. **Arua Yolam:** data curation, investigation, methodology, writing – review and editing. **Thibaud Gruber:** resources, writing – review and editing. **Catherine Hobaiter:** writing – review and editing, resources, methodology, supervision. **Gal Badihi:** writing – review and editing, supervision, conceptualization, methodology, writing – original draft, validation.

## Supporting information


**Audio S1:** Chuckle (i.e., staccato bark) produced by an adult individual of unknown sex during a resting context

**Audio S2:** Grunts produced by an adult male during a resting context

**Audio S3:** Scream produced by an adult individual of unknown sex during a moving context

**Audio S4:** Whoop‐gobble produced by an individual of unknown sex and age in an unknown context

**Audio S5:** Half‐chuckle produced by an individual of unknown sex and age in an unknown context

Supporting File

## Data Availability

The data that support the findings of this study are available from the corresponding author upon reasonable request.
