# Supplementary material for: The Vocal Repertoire of Gray‐Cheeked Mangabeys (Lophocebus albigena) in the Bugoma Forest Reserve, Uganda
Source: Am J Primatol. 2026 Sep 2;88(9):e70204. doi: 10.1002/ajp.70204 (PMC13535742; doi:10.1002/ajp.70204)
Supplement: Supplementary file 1 [file AJP-88-e70204-s003.docx]

**Table S1:** VIF values assessing multicollinearity among acoustic measures

| **Dependent variable:** | **Duration** | **F0 mean** | **F0 range** | **Mean duration el.** | **Number el.** |
| --- | --- | --- | --- | --- | --- |
| **Duration** | NA | 3.841 | 3.826 | 1.476 | 1.372 |
| **F0 mean** | 3.454 | NA | 1.093 | 3.45 | 3.414 |
| **F0 range** | 3.472 | 1.102 | NA | 3.504 | 3.506 |
| **Mean dur. el.** | 1.107 | 2.879 | 2.898 | NA | 1.305 |
| **Number el.** | 1.113 | 3.08 | 3.135 | 1.411 | NA |

**Supplementary Analysis S1:** DFA with equal priors probabilities

The discriminant function analysis (DFA) revealed that the four call types are acoustically distinct, allowing for their successful classification based on acoustic features. The two main discriminant canonical variates extracted by the DFA accounted for 54,5% (LDA1) and 43,1% (LDA2) of the discriminatory power, respectively (hence, reaching 97.6% of the between group variability explained in total). The projection of vocalizations onto these two axes shows a more or less clear separation between the four call categories (**Figure S1**).

The first canonical variate (LDA1), which accounts for most of the discriminatory power, was primarily driven by the mean duration of elements and the total duration of the calls, both of which loaded negatively (-2.6166 and -0.4749, respectively). This axis therefore reflected a temporal dimension, with longer vocalizations (such as whoop-gobbles) on one end and shorter, more fragmented ones on the other.

The second canonical variate (LDA2) was mainly influenced by the mean duration of elements (-0.8154). This dimension helped to distinguish screams, which were positioned on the negative side of the axis.

**Figure S1:** Projection of vocalizations onto the first two canonical variates (LDA1 and LDA2);

each point represents a call

The coefficients of the linear discriminants for each acoustic variable in the first two canonical variates (LDA1 and LDA2) are presented in **Table S2**.

**Table S2:** Coefficients of the linear discriminants for each acoustic variables in LDA1 and LDA2

| Variable | LDA1 | LDA2 |
| --- | --- | --- |
| F0 mean | 0.0007 | -0.0022 |
| F0 range | 0.0001 | -0.0001 |
| Duration | -0.4749 | -0.1266 |
| Number elements | 0.1855 | 0.0729 |
| Mean duration elements | -2.6166 | -0.8154 |

The performance of the model was evaluated using leave-one-out cross-validation, with equal prior probabilities assigned to the four call types. The overall correct classification rate obtained was 92.8% (95% confidence intervals = 89.8-97.0%, bias = 0.99%, standard error = 1.6%), indicating high precision even within the limited sample of whoop-gobbles. The confusion matrix resulting from this cross-validation is presented in **Table S3**.

Most chuckles and screams were correctly classified, and all whoop-gobbles achieved a 100% success rate. However, some grunts were misclassified as chuckles, which represents the main source of classification error (n = 9).

**Table S3:** Cross-validated confusion matrix obtained through leave-one-out cross-validation

|  | Chuckle | Grunts | Scream | Whoop-gobble | Sum |
| --- | --- | --- | --- | --- | --- |
| Chuckle | 71 | 0 | 0 | 1 | **72** |
| Grunts | 9 | 30 | 0 | 0 | **39** |
| Scream | 2 | 0 | 36 | 0 | **38** |
| Whoop-gobble | 0 | 0 | 0 | 18 | **18** |
| Sum | **82** | **30** | **36** | **19** | **167** |

**Table S4:** Standardized residuals for call type–activity associations

|  | Chuckle | Grunts | Scream | Whoop-gobble |
| --- | --- | --- | --- | --- |
| Allogrooming | -3.0 | 3.4 | -0.7 | -0.5 |
| Feeding | -1.4 | 2.2 | -2.4 | -1.2 |
| Moving | 0.8 | -1.3 | 1.7 | 0.3 |
| Resting | 2.5 | -2.6 | -0.7 | 1.3 |
| Social affiliative | -1.6 | 1.7 | -0.4 | -0.2 |
| Social agonistic | -1.6 | -1.6 | 14.3 | -0.3 |

**Table S5:** Standardized residuals for call-participation associations

|  | Chuckle | Grunts | Scream | Whoop-gobble |
| --- | --- | --- | --- | --- |
| Chorus | -10.5 | 11.5 | -2.8 | -2.9 |
| Exchange | 11.5 | -10.6 | -3.1 | -1.2 |
| Single | -1.3 | 0.1 | 3.9 | 2.6 |

**Table S6:** Standardized residuals for call participation-distance associations

|  | Chorus | Exchange |
| --- | --- | --- |
| <25m | 8.8 | -7.7 |
| 25-50m | -1.9 | 1.6 |
| 51-100m | -7.4 | 6.4 |
| 101-150m | -4.9 | 4.2 |
| 151-200m | -2.6 | 2.3 |
| 201-250m | -1.9 | 1.6 |
| >250m | -2.3 | 2.0 |

**Table S7:** Description of call subtypes and their acoustic characteristics

| Call type | Subtype | Description |
| --- | --- | --- |
| Chuckle | Chuckle | Call made of 2 elements, chained with a short pause; each element is composed of several notes |
|  | Half-chuckle | Call made of a single element composed of several notes |
|  | Chuckle variant | Call made of several elements (more than 2), chained with a short pause; each element is composed of several notes |
| Grunt | Grunt | Short, low-frequency call, typically produced in series |
|  | Soft grunt | Same type of unit as the normal grunt but with lower overall intensity |
|  | Grunt train | A sequence of multiple grunts emitted in close succession in which intensity progressively increases over the course of the sequence |

**Figure S2:** Illustration of an initial chuckle note with higher pitch on a spectrogram of a

chuckle produced by an unidentified individual
